# The Role of PSA Density among PI-RADS v2.1 Categories to Avoid an Unnecessary Transition Zone Biopsy in Patients with PSA 4-20 ng/mL

**DOI:** 10.1155/2021/3995789

**Published:** 2021-10-11

**Authors:** Zhi-bing Wang, Chao-gang Wei, Yue-yue Zhang, Peng Pan, Guang-cheng Dai, Jian Tu, Jun-kang Shen

**Affiliations:** ^1^Department of Radiology, The Second Affiliated Hospital of Soochow University, Suzhou 215004, China; ^2^Department of Radiology, Huai'an Hospital of Huai'an City, Huai'an 223200, China; ^3^Department of Urology, The Second Affiliated Hospital of Soochow University, Suzhou 215004, China; ^4^Department of Pathology, The Second Affiliated Hospital of Soochow University, Suzhou 215004, China

## Abstract

**Objective:**

To evaluate the role of prostate-specific antigen density (PSAD) in different Prostate Imaging Reporting and Data System version 2.1 (PI-RADS v2.1) categories to avoid an unnecessary biopsy in transition zone (TZ) patients with PSA ranging from 4 to 20 ng/mL.

**Materials and Methods:**

In this retrospective and single-center study, 333 biopsy-naïve patients with TZ lesions who underwent biparametric magnetic resonance imaging (bp-MRI) were analyzed from January 2016 to March 2020. Multivariate logistic regression analyses were performed to determine independent predictors of clinically significant prostate cancer (cs-PCa). The receiver operating characteristic (ROC) curve was used to compare diagnostic performance.

**Results:**

PI-RADS v2.1 and PSAD were the independent predictors for TZ cs-PCa in patients with PSA 4-20 ng/mL. 0.9% (2/213), 10.0% (7/70), and 48.0% (24/50) of PI-RADS v2.1 score 1-2, 3, and 4-5 had TZ cs-PCa. However, for patients with PI-RADS v2.1 score 1-2, there were no obvious changes in the detection of TZ cs-PCa (0.8% (1/129), 1.3% (1/75), and 0.0% (0/9)) combining with different PSAD stratification (PSAD < 0.15, 0.15-0.29, and ≥0.30 ng/mL/mL). For patients with PI-RADS v2.1 score ≥ 3, the TZ cs-PCa detection rate significantly varied according to different PSAD stratification. A PI-RADS v2.1 score 3 and PSAD < 0.15 and 0.15-0.29 ng/mL/mL had 8.6% (3/35) and 3.7% (1/27) of TZ cs-PCa, while a PI-RADS v2.1 score 3 and PSAD ≥ 0.30 ng/mL/mL had a higher TZ cs-PCa detection rate (37.5% (3/8)). A PI-RADS v2.1 score 4-5 and PSAD <0.15 ng/mL/mL had no cs-PCa (0.0% (0/9)). In contrast, a PI-RADS v2.1 score 4-5 and PSAD 0.15-0.29 and ≥0.30 ng/mL/mL had the highest cs-PCa detection rate (50.0% (10/20), 66.7% (14/21)). It showed the highest AUC in the combination of PI-RADS v2.1 and PSAD (0.910), which was significantly higher than PI-RADS v2.1 (0.889, *P* = 0.039) or PSAD (0.803, *P* < 0.001).

**Conclusions:**

For TZ patients with PSA 4-20 ng/mL, PI-RADS v2.1 score ≤ 2 can avoid an unnecessary biopsy regardless of PSAD. PI-RADS v2.1 score ≥ 3 may avoid an unnecessary biopsy after combining with PSAD. PI-RADS v2.1 combined with PSAD could significantly improve diagnostic performance.

## 1. Introduction

Serum prostate-specific antigen (PSA) screening has been widely used for detecting an early stage of prostate cancer (PCa), evaluating treatment response, and determining tumor progression. However, PSA testing increases the risk of overdiagnosis due to the low specificity, resulting in unnecessary prostate biopsies. In addition, the PCa detection rate varies from the PSA range. Several studies have proven that cancer detection rates were 11.8-20.5%, 20.5-25.0%, and 47.1-53.0% in the PSA range of 4-10 ng/mL, 10-20 ng/mL, and greater than 20 ng/mL, respectively [1–3]. Compared with that in the PSA level of 4-10 ng/mL (defined as “grey zone”), the cancer detection rate in patients with the PSA level of 10-20 ng/mL was not significantly different [2, 4]. Hence, it is feasible to focus on this study in the range of the PSA level from 4 to 20 ng/mL. To reduce unnecessary prostate biopsies, these clinical indicators, including age, prostate volume (PV), PSA density (PSAD), and free to total PSA ratio (f/t-PSA), were used to improve the ability of PCa detection, especially in the PSA range of 4-20 ng/mL.

Multiparametric magnetic resonance imaging (mp-MRI) offers increasingly reliable visualization for the diagnosis of PCa, especially for clinically significant PCa (cs-PCa, defined as Gleason score ≥ 7 and/or volume ≥ 0.5 cm^3^ and/or extra prostatic extension), and provides information for evaluating tumor staging and monitoring treatment response [5]. To improve the accuracy of performance and reporting standardization of prostate mp-MRI examination, the original version of the Prostate Imaging Reporting and Data System (PI-RADS v1) was published in 2012 [6] and then updated as PI-RADS v2 in 2014 [7]. However, PI-RADS v2 had some limitations, especially ambiguous description for typical and atypical nodules in the transition zone (TZ) of the prostate, making it necessary to release the latest version of PI-RADS (PI-RADS v2.1) in 2019 [8]. In addition, compared with the mp-MRI protocol, an abbreviated biparametric MRI (bp-MRI) protocol consisting solely of T2-weighted imaging (T2WI) and diffusion-weighted imaging (DWI) had comparable diagnostic performance for PCa. Meanwhile, it could shorten MRI examination time, reduce the cost, and avoid potential contrast-associated risks [9–11].

Most PCa tumors originate in the peripheral zone (PZ) of the prostate; approximately 25% of these cancers arise from TZ [12]. TZ PCa is more challenging to detect and diagnose on MRI due to these mimics, including benign prostatic hyperplasia (BPH) stromal nodules, chronic prostatitis, or other conditions [13]. Additionally, EAU guidelines mention that TZ sampling during baseline biopsies has a low detection rate and should be limited to MRI-detected lesions or repeat biopsies [14]. A recent study from Byun et al. [15] has revealed that PI-RADS v2.1 shows a better diagnostic performance (sensitivity, 94.5% vs. 91.8%; specificity, 60.9% vs. 56.3%) and a higher interreader agreement (kappa value, 0.565 vs. 0.534) for the detection of TZ cs-PCa compared with PI-RADS v2. Similar results were reported by Tamada et al. [16]. However, the diagnostic specificity, positive predictive value, and accuracy using PI-RADS v2.1 alone had only 50.0%-56.3%, 61.0%-63.2%, and 70.7%-72.4% [16].To date, several risk calculators, a few including MRI and clinical indicators, have been developed and validated. MRI improves accuracy of each of the currently available risk calculators [17]. Hence, it is necessary to combine the PI-RADS v2.1 score with clinical indicators in order to better improve the diagnostic performance for TZ cs-PCa. Most studies have focused on the diagnostic value of combining PI-RADS v2 with clinical indicators and demonstrated that adding these clinical indicators to the PI-RADS v2 score could improve diagnostic performance for the assessment of PCa or cs-PCa [18–20]. A recent study from Han et al. has compared the diagnostic performance of bp-MRI and mp-MRI using PI-RADS v2.1 combined with PSAD in detecting cs-PCa patients with PSA 4-10 ng/mL [21]. Wei et al. [22] have constructed a novel internally validated nomogram based on PI-RADS v2.1 and PSAD to predict TZ cs-PCa. However, the clinical importance of PSAD must be different among PI-RADS categories 1 to 5. To our knowledge, no related studies have been reported on the impact of PSAD stratification on the combined diagnosis based on the PI-RADS v2.1 category.

Therefore, this study is to evaluate the role of PSAD in different PI-RADS v2.1 categories to avoid an unnecessary biopsy in TZ patients with PSA levels of 4-20 ng/mL.

## 2. Materials and Methods

### 2.1. Patient Cohort

**Figure 1:**
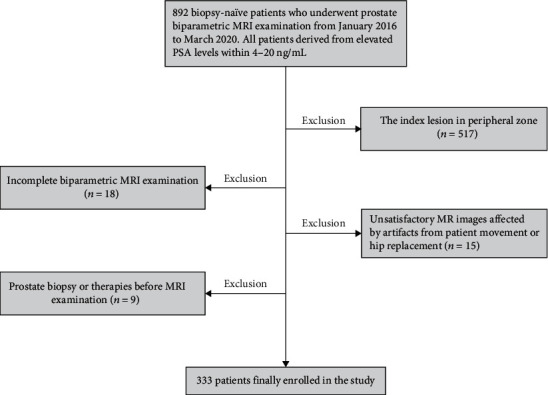
Flowchart of the study population with exclusion criteria.

This retrospective and single-center study was approved by our institutional review board who waived the requirement for the informed consent. We evaluated 892 biopsy-naïve patients who underwent bp-MRI examination between January 2016 and March 2020 at our institution (university hospital). All patients derived from elevated PSA levels within 4-20 ng/mL. Patients who had the index lesion in TZ which was determined as the lesion with the highest PI-RADS v2.1 score were included in the study. For patients with multiple lesions in both TZ and PZ, only the index lesion with the highest score in TZ was included. However, 559 patients were excluded based on these criteria, including (a) the index lesion in PZ (*n* = 517), (b) incomplete bp-MRI examination (*n* = 18), (c) unsatisfactory MR images affected by artifacts from patient movement or hip replacement (*n* = 15), and (d) prostate biopsy or therapies before MRI examination (*n* = 9). Finally, the remaining 333 patients were enrolled. The study population flowchart is shown in Figure 1.

### 2.2. MRI Techniques

bp-MRI examinations were performed on a 3.0 Tesla MRI (Philips Ingenia, the Netherlands) with a 32-channel body phased-array coil. The MR image acquisition protocol was as follows: axial T2WI sequence (repetition time (TR), 3000 ms; echo time (TE), 100 ms; slice thickness, 3 mm; no slice gap; field of view (FOV), 220 × 220 mm) and sagittal T2WI sequence (TR, 4978 ms; TE, 100 ms; slice thickness, 1.5 mm; slice gap, 0.15 mm; FOV, 240 × 180 mm). The axial DWI sequence (TR, 6000 ms; TE, 77 ms; slice thickness, 3 mm; no slice gap; FOV, 260 × 260 mm) had multiple *b* values (*b* = 0, 100, 1000, 2000 s/mm [2]). Apparent diffusion coefficient (ADC) maps were obtained from *b* = 100 and *b* = 1000 s/mm^2^.

### 2.3. Imaging Interpretation and Clinical Data Analysis

All bp-MRI images were independently evaluated by two experienced readers (reader 1 with 5 years of experience in the genitourinary system; reader 2 with 8 years of experience in the genitourinary system) using the PI-RADS v2.1 category protocol in the same setting. They were blinded to pathological results and clinical information. Any discordance between the two readers was resolved by consensus, and any continuous disagreement in scoring was resolved by a third senior urogenital radiologist who made the final determination of the PI-RADS v2.1 score. According to the PI-RADS v2.1 protocol [8], the T2WI sequence plays a primary role for evaluating TZ lesions, while the DWI sequence is assigned a secondary role.

Clinical information including age, t-PSA, f/t-PSA, PV, and PSAD was collected and measured. According to the PI-RADS v2.1 protocol, maximum longitudinal diameter (LD) and maximum anteroposterior diameter (APD) should be measured on the midsagittal T2WI, whereas maximum transverse diameter (TD) should be measured on the axial T2WI. PV was calculated using the following formulation: PV = (maximum APD) × (maximum TD) × (maximum LD) × 0.52 [8]. PSAD was obtained from the t-PSA level divided by the PV (PSAD = t‐PSA/PV) [23].

### 2.4. Reference Standard

In our institution, all patients with PSA 4 ng/mL or higher did undergo biopsy as the standard of care. All biopsy-naïve patients in this study underwent a 10-core systematic transrectal ultrasound- (TRUS-) guided prostate biopsy. The 10-core biopsies were obtained from the base (2 cores), midgland (2 cores), and apex (1 core) from each side of the prostate. Patients with negative MRI (PI-RADS v2.1 scores 1 and 2) underwent a standard 10-core systematic TRUS-guided prostate biopsy only. For suspicious PCa lesions on MRI (PI-RADS v2.1 score ≥ 3), an MRI-TRUS fusion-guided targeted biopsy was used, and then, 2-3 targeted cores would be added for these lesions. The MRI-TRUS fusion-guided targeted biopsy was performed using Esaote's MyLab Twice ultrasound system (Esaote, Italy). Histopathologic evaluation of the biopsy specimens was analyzed and reported by experienced genitourinary pathologists in our institution according to the International Society of Urological Pathology (ISUP) 2014 updated Gleason score grading system [24, 25]. A Gleason score ≥ 7 on the MRI-TRUS fusion targeted biopsy and/or a matching segment on a systematic TRUS-guided prostate biopsy was considered positive for cs-PCa.

### 2.5. Statistical Analysis

Statistical analysis was performed using SPSS 22.0 software and MedCalc version 15.2.2. The Kolmogorov-Smirnov test or Shapiro-Wilk test and Levene's test were used to assess the normality of data and the homogeneity of variances. The independent-sample *t*-test was used for continuous variables which were normally distributed and expressed as mean and standard deviation (SD). Those continuous variables with nonnormal distribution or categorical variables were analyzed using the Mann-Whitney *U* test, presented as median and interquartile ranges. Multivariable logistic regression analysis was performed to determine the independent predictors. The areas under the receiver operating characteristic curve (AUC) with corresponding 95% confidence intervals (CI) were calculated, and DeLong's test was used to compare the diagnostic performance. A two-tailed *P* value less than 0.05 was considered statistically significant.

## 3. Results

### 3.1. Patient Demographics

The characteristics of all patients are shown in Table 1. Totally, 333 patients were enrolled in this study, including 66 (19.8%) cases of PCa and 267 (80.2%) cases of noncancerous lesions. Within these PCa patients, there were 33 (9.9%) with cs-PCa (ISUP ≥ 2) and 33 (9.9%) with low-risk PCa (ISUP 1). A total of 267 noncancerous patients had 193 with benign prostatic hyperplasia and 74 with acute or chronic prostatitis. For the comparison of cs-PCa with low-risk PCa and non-PCa lesions in TZ patients with PSA 4-20 ng/mL, the differences in the age, t-PSA, f/t-PSA, PSAD, PV, and PI-RADS v2.1 score were statistically significant (*P* < 0.05), while the f-PSA was not significantly different (*P* = 0.898).

### 3.2. Multivariate Logistic Regression Analysis

These univariate indicators (age, t-PSA, f/t-PSA, PSAD, PV, and PI-RADS v2.1 score) were significantly different between patients in PSA 4-20 ng/mL with and without cs-PCa. However, PSAD was obtained from t-PSA and PV. To better handle strongly correlated variables, the multivariate model removed t-PSA and PV with smaller AUCs (AUC_t‐PSA_ = 0.638, AUC_PV_ = 0.207) compared with PSAD (AUC_PSAD_ = 0.832). So the final indicators include age, f/t-PSA, PSAD, and PI-RADS v2.1 score. As shown in Table 2, multivariate logistic regression analysis demonstrated that the independent predictors for TZ cs-PCa in patients with PSA 4-20 ng/mL were PSAD and PI-RADS v2.1 score (*P* < 0.05).

### 3.3. Detection of cs-PCa in TZ Patients with PSA 4-20 ng/mL

**Figure 2:**
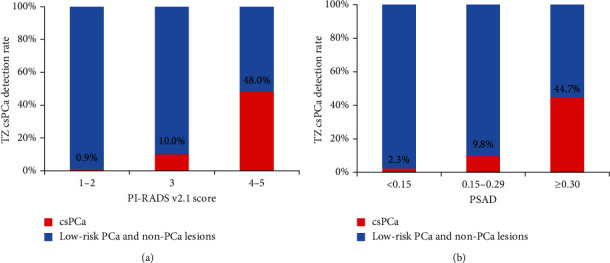
Percentage bar charts for the detection rate of clinically significant prostate cancer in transition zone patients with PSA 4-20 ng/mL stratified by PI-RADS v2.1 score (a) or PSAD (b). Red bar represents clinically significant prostate cancer. Blue bar indicates low-risk prostate cancer (ISUP 1 (Gleason score 3 + 3)) and nonprostate cancer lesions.

**Figure 3:**
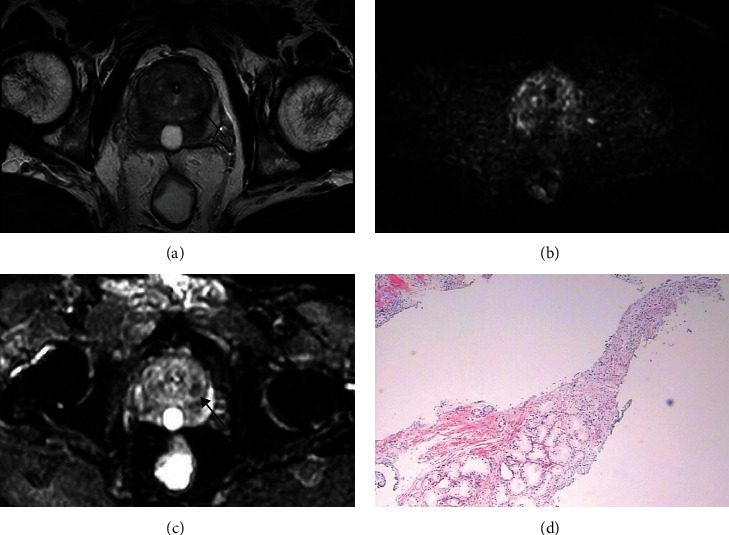
Biparametric MRI of a 65-year-old man with serum total PSA level of 15.18 ng/mL. (a) T2WI showed a homogeneous hypointense mostly encapsulated nodule (arrow) in the left lateral part of TZ; (b) DWI showed no markedly hyperintense signal; (c) ADC map showed a focal lesion with a moderately hypointense signal (arrow). The lesion was assigned a T2WI score of 2, DWI/ADC map scores of 3, and overall score of 2 according to the PI-RADS v2.1 protocol. (d) Pathological image (HE staining) showed that the left TZ lesion was clinically significant prostate cancer (Gleason score 4 + 4).

**Figure 4:**
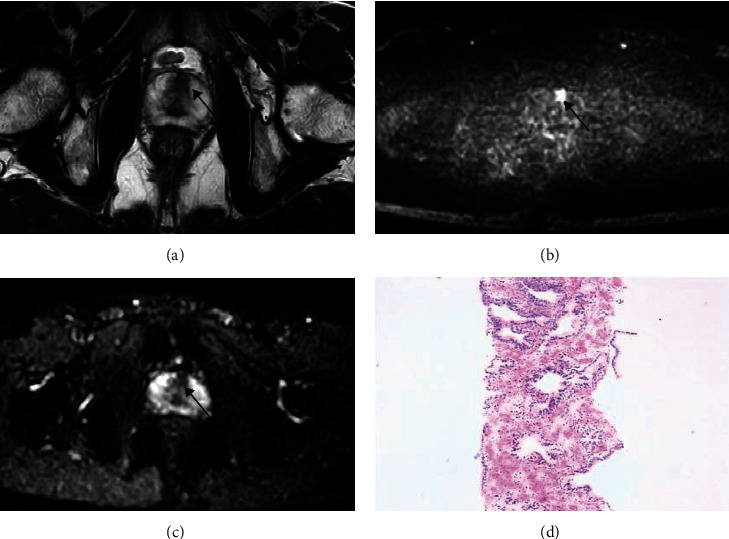
Biparametric MRI of a 54-year-old man with serum total PSA level of 6.04 ng/mL. (a) T2WI showed a homogeneous and moderately hypointense lesion in the anterior part of TZ with the diameter of 12 mm (arrow); (b, c) DWI/ADC map showed focal markedly diffused restriction (arrow). The lesion was assigned a T2WI score of 4, DWI/ADC map scores of 4, and overall score of 4 according to the PI-RADS v2.1 protocol. (d) Pathological image (HE staining) showed that the anterior part of TZ was benign prostate tissue.

The cs-PCa detection rate in TZ patients with PSA 4-20 ng/mL stratified by PI-RADS v2.1 score or PSAD is shown in Figure 2, Table 3. When the PI-RADS v2.1 score was used for the assessment of cs-PCa in TZ patients with PSA levels of 4-20 ng/mL, some cases of misdiagnosis and missed diagnosis inevitably occurred (Figures 3 and 4). Of the 333 patients, 213 (64.0%), 70 (21.0%), and 50 (15.0%) patients were categorized with PI-RADS 1-2, 3, and 4-5, respectively. Of the 213 patients with the PI-RADS score of 1 or 2, only 2 patients (0.9%) had cs-PCa. Of the 70 patients with PI-RADS score 3, 7 patients (10.0%) had cs-PCa. Of the 50 patients with PI-RADS score 4 or 5, 24 patients (48.0%) were diagnosed with cs-PCa. The 333 patients were also stratified according to PSAD levels as <0.15 ng/mL/mL, 0.15-0.29 ng/mL/mL, and ≥0.30 ng/mL/mL. The cs-PCa detection rate in TZ patients with PSA 4-20 ng/mL was 2.3% (4/173) for PSAD < 0.15 ng/mL/mL, 9.8% (12/122) for PSAD 0.15-0.29 ng/mL/mL, and 44.7% (17/38) for PSAD ≥ 0.30 ng/mL/mL, respectively.

Table 4 shows the detection rate of cs-PCa in TZ patients with PSA levels of 4-20 ng/mL combining the PI-RADS v2.1 score (1–3, 4-5) and PSAD (<0.15, 0.15-0.29, and ≥0.30 ng/mL/mL). The cs-PCa detection rate was 0.8%, 1.3%, and 0.0% for PSAD < 0.15, 0.15-0.29, and ≥0.30 ng/mL/mL in patients with PI-RADS v2.1 score ≤ 2. The cs-PCa detection rate was 8.6% for PSAD < 0.15 ng/mL/mL and 3.7% for PSAD 0.15-0.29 ng/mL/mL in patients with PI-RADS v2.1 score 3. A PI-RADS v2.1 score > 3 and PSAD < 0.15 ng/mL/mL had no cs-PCa. In contrast, PI-RADS v2.1 score 3 and PSAD ≥ 0.30 ng/mL/mL yielded 37.5% cs-PCa. A PI-RADS v2.1 score > 3 and PSAD ≥ 0.15 ng/mL/mL yielded 50.0-66.7% cs-PCa, but a PI-RADS v2.1 score > 3 and PSAD ≥ 0.30 ng/mL/mL had the highest cs-PCa detection rate (66.7%).

### 3.4. Diagnostic Performance

**Figure 5:**
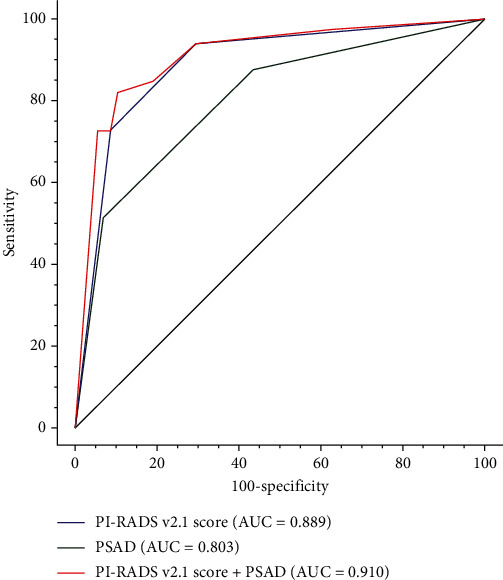
Comparison of the area under ROC curve (AUC) for the assessment of cs-PCa in TZ patients with PSA levels of 4-20 ng/mL based upon the PI-RADS v2.1 score (blue), PSAD (green), and PI-RADS v2.1 score+PSAD (red).

As shown in the ROC curve and DeLong's test (Figure 5**)**, the combination of the PI-RADS v2.1 score and PSAD had the highest AUC for TZ cs-PCa in patients with PSA levels of 4-20 ng/mL (AUC = 0.910 (0.874-0.939)), which was significantly larger than that in the PI-RADS v2.1 score (AUC = 0.889 (0.850-0.920), *P* = 0.039) or PSAD (AUC = 0.803 (0.756-0.844), *P* < 0.001). These results indicated that integrating PSAD and PI-RADS v2.1 score could significantly improve the diagnostic performance for TZ cs-PCa in patients with PSA 4-20 ng/mL.

## 4. Discussion

Serum PSA has been the most widely used for PCa screening and early detection. However, the detection rate of PCa in patients with PSA levels of 4-10 and 10-20 ng/mL was low, and the rate of detecting cs-PCa was even lower. A previous study reported that the PCa detection rate was 11.8% and 20.5% in the PSA range of 4-10 and 10-20 ng/mL [1]. In addition, the PSA level and the prevalence of PCa varied among races, and the PSA “gray zone” range in Asian men should be higher than 4-20 ng/mL compared to the traditional gray zone (4-10 ng/mL) [2, 26]. In this study, we found that the detection rate of cs-PCa in TZ patients with PSA levels ranging from 4 to 20 ng/mL was only 9.9% (33/333). It is more challenging to accurately diagnose TZ lesions in patients with PSA 4-20 ng/mL. Thus, there is a need to reduce an unnecessary prostate biopsy and improve the cancer detection rate for patients with PSA 4-20 ng/mL.

In the present study, our results found that the PI-RADS v2.1 score was one of the two independent predictors for TZ cs-PCa in patients with PSA 4-20 ng/mL. Compared to PI-RADS v2, the PI-RADS v2.1 made small step modifications to simplify MRI interpretation and improve interreader agreement [8, 27]. Some studies demonstrated that PI-RADS v2.1 yielded higher diagnostic performance and interreader agreement among readers of various experiences for the detection of TZ PCa or cs-PCa than PI-RADS v2 [28–30]. In addition, PI-RADS v2.1 also recommended a bp-MRI protocol using T2WI and DWI, while eliminating DCEI, and should be encouraged to clinical researches [8]. The PI-RADS v2.1 category in our study was performed under the bp-MRI protocol.

For the detection of cs-PCa in TZ patients with PSA 4-20 ng/mL, we found that the cs-PCa detection rate was 0.9% (2/213) for PI-RADS v2.1 score ≤ 2. There was no significant change in the detection rate of cs-PCa after combining with PSAD, which indicated that for patients with PSA levels of 4-20 ng/mL, PI-RADS v2.1 score ≤ 2 can avoid an unnecessary biopsy regardless of the PSAD value. For PI-RADS v2.1 score 3, the cs-PCa detection rate was 10.0% (7/70). However, combining with PSAD < 0.15 ng/mL/mL (8.6%, 3/35) or PSAD 0.15-0.29 ng/mL/mL (3.7%, 1/27) can avoid an unnecessary biopsy. Importantly, for PI-RADS v2.1 score > 3, patients with PSAD < 0.15 ng/mL/mL (0.0%, 0/9) can also avoid an unnecessary biopsy. In addition, compared to the PI-RADS v2.1 score, the cs-PCa detection rate was the highest (66.7%) for the combination of PI-RADS v2.1 score > 3 and PSAD ≥ 0.30 ng/mL/mL. Our results were substantially consistent with the previous PI-RADS v2.0 study by Ryoo et al. [31]. They concluded that patients with PI-RADS v2 score ≤ 2 may avoid an unnecessary biopsy regardless of PSAD; patients with PI-RADS v2 score 3 may avoid an unnecessary biopsy through PSAD results. Washino et al. [18] also reported similar results that patients with PI-RADS v2 score ≤ 3 and PSAD < 0.15 ng/mL/mL may avoid an unnecessary biopsy. In contrast, the combination of PI-RADS v2 score > 3 and PSAD ≥ 0.15 ng/mL/mL or PI-RADS v2 score 3 and PSAD ≥ 0.30 ng/mL/mL yielded the highest cs-PCa detection.

Our results also demonstrated that PSAD was the other independent predictor for the assessment of TZ cs-PCa in patients with PSA levels of 4-20 ng/mL. PSAD could distinguish patients with a PSA elevation due to BPH and prostatic inflammation, and it has been proven to improve the accuracy of PI-RADS in the decision to perform prostate biopsies [32]. In most studies, the PSAD cutoff value was set from 0.10 to 0.30 ng/mL/mL. In the present study, we stratified the PSAD value as three groups (<0.15, 0.15-0.29, and ≥0.30 ng/mL/mL). For TZ patients with PSA 4-20 ng/mL, the combination of the PI-RADS v2.1 score and PSAD yielded higher cs-PCa detection rates compared to the PI-RADS v2.1 score. PI-RADS v2.1 score 3 and PSAD ≥ 0.30 ng/mL/mL had a higher cs-PCa detection rate than PI-RADS v2.1 score 3 (37.5% vs. 10.0%). A PI-RADS v2.1 score > 3 and PSAD ≥ 0.15 ng/mL/mL had higher cs-PCa detection rates than PI-RADS v2.1 score > 3 (50.0%-66.7% vs. 48.0%). For PI-RADS v2.1 score 3, the cs-PCa detection rate was higher for PSAD < 0.15 ng/mL/mL (8.6%, 3/35) than that for PSAD 0.15-0.29 ng/mL/mL (3.7%, 1/27). This may be due to sample error caused by the small number of positive cases. In addition, we found that the combination of the PI-RADS v2.1 score and PSAD had better diagnostic performance (AUC = 0.910) than the PI-RADS v2.1 score (AUC = 0.889, *P* = 0.039) or PSAD value (AUC = 0.803, *P* < 0.001), which was comparable with the previous study by Han et al. [21]. They concluded that the combination model of the bp-MRI-based PI-RADS v2.1 score and PSAD had higher diagnostic performance with an AUC of 0.907 compared to the PI-RADS v2.1 score with an AUC of 0.884 and PSAD with an AUC of 0.682. The different AUC of PSAD may be due to differences in patient characteristics, i.e., PSA ranges from 4 to 20 ng/mL and TZ cs-PCa in the present study and PSA ranges 4-10 ng/mL and total cs-PCa (PZ and TZ) in the previous study. These results indicated that the combination of PI-RADS v2.1 and PSAD could significantly improve the diagnostic performance for TZ cs-PCa in patients with PSA 4-20 ng/mL.

Several limitations need to be considered in this study. First, there was a retrospective, single-center study with relatively small number of patients, so sample selection bias inevitably exists. The present results would be further validated in multicenter studies with a larger number of patients. Second, our results were performed based on a “patient” level who had the index lesion with the highest PI-RADS v2.1 score among all lesions in TZ, not on a “lesion” level. Third, not all patients underwent an MRI-TRUS fusion-guided targeted biopsy, and those potential PCa lesions with MRI-negative and pathology-positive status may be missed.

## 5. Conclusions

For TZ patients with PSA 4-20 ng/mL, PI-RADS v2.1 score ≤ 2 can avoid an unnecessary biopsy, regardless of PSAD. PI-RADS v2.1 score ≥ 3 may avoid an unnecessary biopsy after combining with PSAD. In addition, PI-RADS v2.1 combined with PSAD could significantly improve its diagnostic performance.

## Figures and Tables

**Table 1:** Patient demographics.

	cs-PCa (*n* = 33)	Low-risk PCa and non-PCa lesions (*n* = 300)	*Z* value	*P*
Age (years)	72 (66-78)	69 (63-74)	-2.234	0.025^∗^
t-PSA (ng/mL)	11.42 (8.26-15.48)	9.27 (6.78-12.59)	-2.607	0.009^∗^
f-PSA (ng/mL)	1.50 (0.99-2.00)	1.41 (0.98-1.96)	-0.128	0.898
f/t-PSA	0.12 (0.09-0.16)	0.15 (0.12-0.20)	-3.002	0.003^∗^
PSAD (ng/mL/mL)	0.31 (0.20-0.42)	0.14 (0.10-0.20)	-6.252	<0.001^∗^
PV (mL)	34.16 (26.47-50.86)	66.12 (47.99-85.46)	-5.526	<0.001^∗^
PI-RADS v2.1 score^#^			-8.018	<0.001^∗^
1	1	43 (3)		
2	1	168 (11)		
3	7	63 (10)		
4	8	18 (6)		
5	16	8 (3)		
ISUP-2014 grading				
ISUP 1	N.A.	33		
ISUP 2	12	N.A.		
ISUP 3	9	N.A.		
ISUP 4	7	N.A.		
ISUP 5	5	N.A.		

t-PSA: total prostate-specific antigen; f-PSA: free prostate-specific antigen; f/t-PSA: free to total PSA ratio; PSAD: prostate-specific antigen density; PV: prostate volume; PCa: prostate cancer; cs-PCa: clinically significant prostate cancer; low-risk PCa: ISUP 1 (Gleason score 3 + 3); PI-RADS v2.1: Prostate Imaging Reporting and Data System, version 2.1; ISUP: International Society of Urological Pathology; N.A.: not applicable. ^#^The number in parentheses shows the number of patients with low-risk PCa. ^∗^*P* < 0.05.

**Table 2:** Multivariate logistic regression for TZ cs-PCa in patients with PSA 4-20 ng/mL.

Parameters	cs-PCa			
	OR	95% CI	*β*	*P*
Age	1.021	0.960-1.086	0.021	0.500
f/t-PSA	0.245	0.000-190.115	-1.408	0.678
PSAD	594.440	11.395-31010.36	6.388	0.002^∗^
PI-RADS v2.1 score				<0.001^∗^
1	Ref			
2	0.190	0.011-3.205	-1.663	0.249
3	3.312	0.382-28.749	1.198	0.277
4	6.893	0.704-67.454	1.930	0.097
5	35.384	3.786-330.724	3.566	0.002

f/t-PSA: free to total PSA ratio; PSAD: prostate-specific antigen density; PI-RADS v2.1: Prostate Imaging Reporting and Data System, version 2.1; cs-PCa: clinically significant prostate cancer; OR: odds ratio; CI: confidence interval. ^∗^*P* < 0.05.

**Table 3:** The detection of TZ cs-PCa in PSA 4-20 ng/mL stratified by PI-RADS v2.1 or PSAD.

	PI-RADS v2.1 score			PSAD		
	1-2 (*n* = 213)	3 (*n* = 70)	4-5 (*n* = 50)	<0.15 (*n* = 173)	0.15-0.29 (*n* = 122)	≥0.30 (*n* = 38)
cs-PCa (*n*)	2	7	24	4	12	17
Low-risk PCa and non-PCa lesions (*n*)	211	63	26	169	110	21
Cancer detection rate (%)	0.9% (2/213)	10.0% (7/70)	48.0% (24/50)	2.3% (4/173)	9.8% (12/122)	44.7% (17/38)

PSAD: prostate-specific antigen density; cs-PCa: clinically significant prostate cancer; low-risk PCa: ISUP 1 (Gleason score 3 + 3); PI-RADS v2.1: Prostate Imaging Reporting and Data System, version 2.1.

**Table 4:** TZ cs-PCa detection rate in PSA 4-20 ng/mL stratified by PI-RADS v2.1 score and PSAD.

		PSAD		
		<0.15	0.15-0.29	≥0.30
PI-RADS v2.1	1-2	0.8% (1/129)	1.3% (1/75)	0.0% (0/9)
	3	8.6% (3/35)	3.7% (1/27)	37.5% (3/8)
	4-5	0.0% (0/9)	50.0% (10/20)	66.7% (14/21)

PSAD: prostate-specific antigen density; PI-RADS v2.1: Prostate Imaging Reporting and Data System, version 2.1.

## Data Availability

The data used to support the findings of this study are available from the corresponding author upon request.

## Abbreviations

ADC: Apparent diffusion coefficient
APD: Anteroposterior diameter
AUC: Area under the curve
BPH: Benign prostatic hyperplasia
bp-MRI: Biparametric magnetic resonance imaging
CI: Confidence interval
cs-PCa: Clinically significant prostate cancer
DWI: Diffusion-weighted imaging
ISUP: International Society of Urological Pathology
LD: Longitudinal diameter
mp-MRI: Multiparametric magnetic resonance imaging
MRI: Magnetic resonance imaging
PCa: Prostate cancer
PI-RADS: Prostate Imaging Reporting and Data System
PSA: Prostate-specific antigen
PSAD: Prostate-specific antigen density
PV: Prostate volume
PZ: Peripheral zone
SPSS: Statistical Package for the Social Sciences
T2WI: T2-weighted imaging
TD: Transverse diameter
t-PSA: Total prostate-specific antigen
TRUS: Transrectal ultrasound
TZ: Transition zone
